# Waist circumference measurement sites and their association with visceral and subcutaneous fat and cardiometabolic abnormalities

**DOI:** 10.20945/2359-3997000000055

**Published:** 2018-08-01

**Authors:** Cláudia Porto Sabino Pinho, Alcides da Silva Diniz, Ilma Kruze Grande de Arruda, Ana Paula Dornelas Leão Leite, Marina de Moraes Vasconcelos Petribu, Isa Galvão Rodrigues

**Affiliations:** 1 Universidade Federal de Pernambuco Universidade Federal de Pernambuco Recife PE Brasil Universidade Federal de Pernambuco (UFPE), Recife, PE, Brasil; 2 Universidade de Pernambuco Universidade de Pernambuco Recife PE Brasil Pronto-Socorro Cardiológico Universitário de Pernambuco, Recife, PE, Brasil

**Keywords:** Waist circumference, visceral fat, subcutaneous fat, abdominal fat

## Abstract

**Objectives:**

To estimate the degree of variability of the waist circumference (WC) when obtained in different anatomical sites and compare the performance of the measurement sites as predictors of visceral adipose tissue (VAT) and subcutaneous adipose tissue (SAT) and cardiometabolic abnormalities.

**Subjects and methods:**

Cross-sectional study involving 119 individuals with overweight (50.3 ± 12.2 years), in which six WC measurement sites were evaluated (minimal waist, immediately below the lowest rib, midpoint between the lowest rib and the iliac crest, 2 cm above the umbilicus, immediately above the iliac crest, umbilicus level), in addition to the VAT and SAT (quantified by computed tomography) and cardiometabolic parameters.

**Results:**

The differences between the measurements ranged from 0.2 ± 2.7 cm to 6.9 ± 6.7 cm for men, and from 0.1 ± 3.7 cm to 10.1 ± 4.3 cm for women. The minimum waist showed significant correlation with VAT (r = 0.70) and with a higher number of cardiometabolic parameters among men. Regarding women, the WC measurement showed high correlation with SAT and moderate correlation with VAT, not being found superiority of one measurement protocol in relation to the others when assessed the correlation with VAT and with cardiometabolic parameters.

**Conclusions:**

Greater variability between the measuring sites was observed among women. With respect to men, the minimum waist performed better as a predictor of VAT and cardiometabolic alterations.

## INTRODUCTION

The waist circumference (WC) measurement has been recommended in clinical guidelines and by the leading authority in health and societies as a cardiometabolic risk predictor associated with central adiposity, both in clinical practice and in epidemiological studies (1-6). Despite the widespread use of this anthropometric parameter, there is no consensus on the evaluation protocol and the measurement standardization due to lack of consistent evidence justifying the superiority of one measurement site in relation to others, resulting in a wide variety of techniques reported in the literature.

A systematic review of 120 studies showed eight different protocols for WC measurement (7), some endorsed by international bodies and other experimental protocols adopted to a lesser extent in the publications 1 on the subject. The World Health Organization (8) and the International Diabetes Federation (4) recommend the measurement at the midpoint between the iliac crest and the lowest rib. The National Institutes of Health (9), in turn, establishes the superior border of the iliac crest as the anatomical site of WC measurement. Other anatomical sites such as the minimal waist and the umbilicus are also commonly adopted (7,10).

Although these measurement sites present close correlation, significant differences between the WC measures obtained at different locations have been reported (11-14). Previous studies comparing the different protocols showed a profound influence of the measurement site on the absolute values of WC (11,13,15). Those differences, even if subtle, could potentially affect the utility of the WC measurement for assessing the cardiometabolic risk, particularly when the stratification of the risk depends on dichotomous thresholds, with possible repercussion on the clinical decision making (16). In addition, little attention has been given to the method for determining the WC when comparing data from different studies (13,14).

This study aimed to estimate the degree of variability of the WC when obtained from different anatomical sites and compare the performance of the measurement sites as predictors of visceral and subcutaneous fat and cardiometabolic abnormalities.

## SUBJECTS AND METHODS

Cross-sectional study developed in a nutrition clinic of a public university hospital, reference in cardiology, in northeastern Brazil, involving individuals with overweight, of both sexes and age ≥ 20 years. In this clinic, the patients are predominantly individuals with non-communicable chronic diseases: obesity, hypertension, diabetes mellitus, metabolic syndrome and dyslipidemia.

Overweight was established based on the body mass index (BMI) ≥ 25 kg/m^2^ for adults (8) and ≥ 27 kg/m^2^ for the elderly (17).

The sample was built based on voluntary adhesion, being picked up patients in first consultation. Individuals with hepatomegaly and/or splenomegaly, ascites and recent abdominal surgery were excluded, as well as pregnant women and those who had children up to 6 months prior to the screening for the study, characteristics that may influence the intra-abdominal fat measurement and/or the anthropometric measurements.

Assuming a 5% α error, a β error of 20%, an estimated average correlation between the WC measures and the metabolic changes of 0.4 (p) and a variability of 0.1 (d^2^), it was obtained a minimum sample size of 108 individuals. To correct possible losses, that number was increased by 10% [100/(100-10)], with a total sample of n = 119.

The visceral adipose tissue (VAT) and the subcutaneous adipose tissue (SAT) were assessed by computed tomography (CT), using the Philips Brilliance CT-10 slice tomoghaph (VMI *Indústria e Comércio Ltda,* Lagoa Santa, MG, Brazil). The survey was conducted in four hours fasting with the patient in supine position. The tomographic cut was obtained with radiographic parameters of 140 kV and 45 mA, at the L4 level, having a thickness of 10 mm. The total area of total abdominal fat and the visceral fat area were manually outlined with free cursor contouring each region. The entire surface of the skin was excluded from the marking area. The area of the VAT was determined using as limits the inner borders of the rectus abdominis, internal oblique and square lumbar muscles, excluding the vertebral body and including the retroperitoneal, mesenteric and omental fat. The subcutaneous fat area was calculated by subtracting the VAT from the total fat area. All areas of fat were described in cm^2^. For identification of the adipose tissue, it was used the density values of −50 and −250 Hounsfield units (18-20).

The WC was obtained by inelastic tape measure, with accuracy of 0.1 cm, directly on the skin, in six anatomical regions: 1) at the minimal waist (narrowest region between the chest and hips) (WC1) (10); 2) immediately below the lowest rib (WC2) (10,11); 3) at the midpoint between the lowest rib and the iliac crest (WC3) (4,8); 4) 2 cm above the umbilicus (WC4) (21); 5) immediately above the iliac crest (WC5) (9); at the umbilicus level (WC6) (7,22).

The bony landmarks of the lowest rib and of the iliac crest were located and palpated by the examiner at the level of the middle axillary line. The measuring tape was placed on a horizontal plane around the abdomen in the locations described above and particular attention was given to ensure that the tape was parallel to the floor and perpendicular to the longitudinal axis of the body. The measurement was performed at the end of the normal expiration with the inelastic tape adjacent to the skin, without compressing it, keeping the participant standing up, straight, with parallel legs and arms hanging down on the sides. For each evaluated anthropometric point, a double measurement was obtained by a trained examiner (12,23). When the measure difference between measurements was greater than 0.1 cm, a third measurement was performed. The final measure considered was the average of the two closest values.

It were evaluated the following cardiometabolic parameters: fasting blood glucose, glycated hemoglobin (HbA1C), lipid profile (triglycerides (TG), total cholesterol (TC) and fractions, non-HDL cholesterol and TG/HDL-c ratio), C-reactive protein (CRP) and uric acid. Samples were collected with 9-12 hours fasting, considering a preparation protocol (24). Blood glucose, lipid profile and uric acid were analyzed by the enzymatic method, and HbA1c and CRP by turbidimetry. Biochemical analyses were carried out using a Cobas Integra 400® analyzer (Roche Diagnostics) in the Laboratory of Clinical Analyses in the service facilities.

The non-HDL cholesterol fraction, calculated by subtracting HDL-c from TC (non-HDL cholesterol = TC - HDL-c), was adopted as the estimate of the total number of atherogenic particles in the plasma (VLDL + IDL + LDL) (24). The TG/HDL-c ratio was used as atherogenicity index for reflecting the size of LDL-c particles (25).

Data were analyzed using the Statistical Package for Social Sciences, version 13.0 (SPSS Inc., Chicago, IL, USA). Continuous variables were tested for normal distribution using the Kolmogorov-Smirnov test, being described as mean and standard deviation or median and interquartile range, according to the distribution pattern. CRP was the only variable that showed non-normal distribution.

The Student *t* test for independent samples was used for comparison of the WC means between the sexes. The one-way ANOVA test was used for comparison of the six WC measurement sites, using the Bonferroni test *a posteriori.* Proportions were compared by Pearson Chi Square.

The Pearson or Spearman correlation was used to evaluate the relationship between the different anatomical sites of WC measurement with VAT, SAT and biochemical parameters. To analyze the correlation between WC and lipid profile, it were excluded subjects who reported use of lipid-lowering medications, and to verify the association between the WC sites and the glycemic parameters (fasting and HbA1c), it were excluded diabetic subjects. Statistical significance was considered when p < 0.05.

## RESULTS

Were included 124 individuals, but after discarding the losses for refusal or information inconsistency (n = 5), 119 patients were included in the final sample of the study. The mean age was 50.3 ± 12.2 years and women predominated (68.1%; CI_95%_: 59.2-75.8). Although men and women have displayed similar characteristics regarding age, nutritional status, subcutaneous fat and prevalence of diabetes mellitus and arterial hypertension, there were higher averages of visceral fat in men (p < 0.001) (Table 1).

**Table 1:** Sample characteristics, stratified by sex (n = 119)

Variables	Males (n = 38)	Females (n = 81)	p-value*
Age, years (mean/SD)	49.9 (±13.7)	50.5 (±11.8)	0.817*
Arterial hypertension (%, CI_95%_)	68.4 (52.5-80.9)	59.3 (47.8-70.0)	0.420§
Diabetes mellitus (%, CI_95%_)	26.3 (15.0-42.0)	21.0 (12.7-31.5)	0.659§
BMI, kg/m^2^ (mean/ SD)	33.1 (±4.9)	33.5 (±5.3)	0.715*
VAT (cm^2^)	378.9 (±118.7)	258.6 (±75.4)	< 0.001*
SAT (cm^2^)	506.3 (±162.2)	540.9 (±145.6)	0.294*

* Student *t* test for independent samples;

§ Pearson Chi Square. SD: standard deviation; CI95%: confidence interval of 95%; BMI: body mass index; VAT: visceral adipose tissue; SAT: subcutaneous adipose tissue.

BMI averages were similar between sexes (p = 0.715), however, WC averages were higher among men, expressing, between sexes, a different distribution pattern of the body fat (Table 2).

**Table 2:** Comparative analysis of the averages of six anatomical sites of measurement of the waist circumference in individuals with overweight, according to sex

Variables	Males (n = 38)	Females (n = 81)	p-value*
WC1 (cm)	106.2 (±10.6)	96.8 (±10.0) cm^a^	< 0.001
WC2 (cm)	107.5 (±11.4)	97.6 (±11.3) cm^a^	< 0.001
WC3 (cm)	112.9 (±12.5)	103.2 (±11.0) cm^b^	< 0.001
WC4 (cm)	112.3 (±11.5)	104.6 (±11.5) cm^b^ ^c^	0.003
WC5 (cm)	109.9 (±13.7)	106.7 (±10.7) cm^c^	0.273
WC6 (cm)	113.1 (±12.5)	106.8 (±10.7) cm^c^	0.012
p-value**	0.143	< 0.001	

* Student *t* Test for independent samples.

** ANOVA *one way.*

a,b,c Different letters mean statistical diferences by the Bonferroni test. WC1: minimal waist; WC2: immediately below the lowest rib; WC3: at the midpoint between the lowest rib and the iliac crest; WC4: 2 cm above the umbilicus; WC5: immediately above the iliac crest; WC6: at the umbilicus level.

It was observed significant difference in the absolute values of the WC obtained in six anatomical sites of women (p < 0.001). Notwithstanding, among men, the difference was not observed (p = 0.143) and may suggest that they have uniformity in the waist circumference along the upper body (Table 2).

The maximum absolute difference among the various protocols examined was observed when compared the WC obtained at the umbilicus (WC6) and at the minimal waist (WC1) in both sexes. The average differences between the measurements ranged from 0.2 ± 2.7 cm to 6.9 ± 6.7 cm among men, and from 0.1 ± 3.7 cm to 10.1 ± 4.3 cm among women.

In men, the minimal waist (WC1) showed better correlation with VAT and lower correlation with subcutaneous fat, unlike other measurement sites, which showed a higher correlation with SAT than with VAT. Furthermore, the minimal waist (WC1) was the only measurement site that showed correlation with triglycerides (r = 0.595, p < 0.05) and with the TG/HDL-c ratio (r = 0.506, p < 0.05).

The measures of the circumferences obtained at the bony landmark of the iliac crest (WC5) and at the umbilicus (WC6) showed higher correlation coefficients with SAT and lower correlation with VAT in both sexes. In women, these two anatomical sites have higher absolute values, expressing that the higher abdominal circumference can be much more represented by the SAT than by the VAT (Table 3).

**Table 3:** Pearson correlation (r) between waist circumference measures obtained at six anatomical sites with visceral and subcutaneous fat and cardiometabolic profile in subjects with overweight, according to sex

Parameters	Males					
	WC1	WC2	WC3	WC4	WC5	WC6
VAT	0.701*	0.598*	0.531*	0.566*	0.358	0.426*
SAT	0.621*	0.700*	0.800*	0.712*	0.836*	0.863*
TC	−0.012	−0.147	−0.197	−0.207	−0.380	−0.300
HDL-c	−0.267	−0.336	−0.421	−0.431	−0.177	−0.415
LDL-c	−0.245	−0.301	−0.316	−0.347	−0.464	−0.401
TG	0.595*	0.425	0.377	0.421	0.139	0.305
Non HDL-c	0.039	−0.082	−0.115	−0.124	−0.345	−0.219
TG/HDL	0.506*	0.324	0.313	0.351	0.012	0.234
Glucose	−0.244	−0.253	−0.243	−0.258	−0.215	−0.264
HbA1C	−0.380	−0.500*	−0.529*	−0.555*	−0.484*	−0.553*
CRP#	−0.131	−0.193	−0.235	−0.230	−0.281	−0.248
Uric acid	0.032	−0.011	−0.022	0.017	−0.183	−0.032
**Parameters**	**Females**					
	**WC1**	**WC2**	**WC3**	**WC4**	**WC5**	**WC6**
VAT	0.462*	0.425*	0.397*	0.383*	0.332*	0.359*
SAT	0.714*	0.732*	0.758*	0.776*	0.783*	0.809*
TC	−0.265	−0.199	−0.198	−0.234	−0.240	−0.241
HDL-c	−0.189	−0.130	−0.126	−0.086	−0.121	−0.110
LDL-c	−0.254	−0.193	−0.199	−0.225	−0.244	−0.229
TG	−0.021	−0.030	−0.037	−0.100	−0.113	−0.143
Non HDL-c	−0.232	−0.177	−0.177	−0.224	−0.222	−0.226
TG/HDL	0.027	0.022	0.032	−0.054	0.000	−0.074
Glucose	0.240	0.251	0.162	0.144	0.131	0.112
HbA1C	0.049	0.032	0.102	−0.012	0.068	0.000
CRP #	0.239	0.205	0.238	0.293*	0.241*	0.246*
Uric acid	0.340*	0.238*	0.313*	0.325*	0.395*	0.362*

* p < 0,05.

# Spearman correlation. VAT: visceral adipose tissue; SAT: subcutaneous adipose tissue; TC: total cholesterol; TG: triglycerides; HbA1C: glycated hemoglobin; CRP: C-reactive protein. WC1: minimal waist; WC2: immediately below the lowest rib; WC3: at the midpoint between the lowest rib and the iliac crest; WC4: 2 cm above the umbilicus; WC5: immediately above the iliac crest; WC6: at the umbilicus level.

It was found, for females, that the six evaluated protocols for obtaining the WC showed similar correlation with VAT (moderate correlation) and with SAT (high correlation). Regarding the metabolic abnormalities, it was observed, in females, that the uric acid correlated directly with the measures obtained at all measurement sites and that the CRP correlated with the three major measures observed among women (WC4, WC5, WC6).

## DISCUSSION

Although much progress has been made with respect to the use of the WC as a predictor of the cardiometabolic risk, up to now there is no ideal and uniform definition for using the WC measurement. One major objective of developing a definition is to minimize measurement errors and thus improve the efficiency in the estimates in studies of association and comparison involving this parameter. Lack of standards regarding anatomical site, posture, breathing phase and other factors contribute to measurement errors. Several protocols have been recommended, but the comparison between them has not been sufficiently explored.

The results of this study indicate that the choice of the measurement protocol influences the magnitude of the WC measurement, especially in women. The substantial differences in the absolute values of the measures obtained for females were not reproduced in males. Similar result was reported by other authors (14,26), who indicated greater impact of the measurement site among women.

These results observed for different genders may reflect sex differences in the abdominal fat distribution pattern. Similar measures among men indicate uniformity in the total fat accumulation deposited throughout the abdomen. Nonetheless, in women, the changes observed in the abdominal girth suggest a more curvilinear structure and with greater accumulation of adiposity in the lower torso.

The maximum differences between the measures observed in this study (6.9 cm for men and 10.1 cm for women) reveal profound influence of the measurement site on the WC value. This result was relatively similar I to the findings reported by Willis and cols. (12), who I showed differences of 4.5 cm and 10.6 cm for males and females, respectively, when evaluating patients with I an average BMI of 30 kg/m^2^. Agarwal and cols. (13) I reported mean differences between the measures of 5.3 cm for men and 5.5 cm for women, when evaluating 123 Asian subjects, with a mean age of 34 ± 8.7 years and average BMI of 23.9 ± 4.9 kg/m^2^. Other studies (23,26) have indicated differences of approximately 2.0 cm for men and 5.5 cm for women. The variability in the differences between the measurement sites reported among diverse populations suggests that these differences may vary depending on the sample characteristics, including age, sex, race/ethnicity and level of adiposity. What seems to be consensus is that the results indicate greater influence of the measurement site on females.

The differences in the measures, even if slight, can have particular effect on the abdominal obesity classification. The abdominal fat as a proxy of the cardiometabolic risk depends on dichotomized thresholds, and subtle variations can exert influence when this risk is stratified. Willis and cols. (12) reported that the estimates of prevalence of abdominal obesity (> 88 cm/> 102 cm) drastically varied according to the measurement site. In women, the measure taken at the minimal waist resulted in lower prevalence (31%) when compared to the measures obtained at the umbilicus (55%). In men, a small average difference between measures (2.5 cm) had a noticeable impact on the prevalence of abdominal obesity (34% when considering the measure taken at the umbilicus and 23% when using the minimal waist). Consequently, the choice of the measurement site can result in significant repercussions on the interpretation of epidemiological data. Therefore, small differences can be amplified when dichotomous cutoff points are used to define abdominal obesity. Despite the risks of using different protocols in the abdominal obesity classification, a systematic review of 120 studies demonstrated similar pattern of association of the different sites of obtention of the WC with cardiovascular diseases, diabetes mellitus and mortality (7).

This study, by engaging a group of individuals with overweight and obesity, did not assess the impact of using different WC evalution protocols on the prevalence of abdominal obesity.

Although some results elect the WC as a better indicator of the visceral fat and of the cardiovascular risk in comparison with the BMI and the waist-hip ratio (WHR) (27-29), few studies (12,23,26) compared the performance of different WC measurement sites as predictors of visceral fat accumulation. Furthermore, the available studies compared the use of only two (12,26) or three measurement protocols (23), different from our study that compared six anatomical sites.

This study showed among men that the minimal waist was a better marker of visceral fat than of subcutaneous fat, unlike other measurement protocols, which showed better correlation with the subcutaneous fat. This observation, coupled with the fact that no significant difference was observed in the WC measures for males, allows us to infer that the visceral fat is more concentrated in the upper abdomen and that VAT and SAT are not evenly disposed over the abdominal wall of males.

Furthermore, the minimal waist was directly correlated with a greater number of cardiometabolic parameters (two) compared to the other measurement sites (one or zero). A similar result was produced by other research (12) that, when evaluating American adults with overweight and obesity, found that the minimal waist showed stronger correlation with VAT (r = 0.64; p < 0.001) and a higher number of metabolic parameters (three) compared to the measure obtained at the umbilicus, which presented lower correlation coefficient with VAT (r = 0.54; p < 0.001) and association with only two metabolic parameters.

It is plausible to suppose that the measure offering the best correlation with VAT would have higher correlation with metabolic changes. It is well established the connection of the visceral obesity with a pro-atherogenic state (30,31) and the epicenter of most of the hypotheses postulated to explain this association (32,33) refers to the portal drainage of VAT, which provides direct access of free fatty acids and adipokines to the liver, by activating immune hepatic mechanisms for the production of inflammatory mediators, and thus promoting greater insulin resistance and increased production of triglycerides (34,35).

Among women, it was found that the WC, regardless of the measurement site, was predominantly a subcutaneous fat index, corroborating the findings of another investigation (23). The minimal waist also showed a slight superiority in the correlation with VAT, when compared to the other measurements, while the larger abdominal circumferences (at the level of the umbilicus or the iliac crest) had lower correlation coefficients. These findings demonstrate that the larger perimeters reflect much more the excess subcutaneous fat than the excess visceral fat and corroborate the indication that the visceral fat would be proportionally more concentrated in the upper abdomen than in the lower abdômen (23,36).

WC reflects the abdominal adipose tissue, not being able to distinguish the deposits of visceral and subcutaneous fat (23,37). However, being used as a cardiometabolic risk predictor, it should better predict the VAT than the SAT, but this is not what the literature has shown (23,26,37), being very important the reflection on the widespread use of WC as a single parameter of risk screening.

Some potential limitations need to be considered when interpreting the data presented. It was not a random sample and the participants of the study were taken from a hospital center which is reference in cardiology. In addition, it were included only individuals with overweight and obesity and therefore the data presented may not be generalized to individuals with low levels of adiposity. The small number of subjects included can also be a limitation in the statistical power of the study and compromise its external validity. Moreover, given that the racial characteristics influence the distribution of body fat, the extrapolation of data for individuals of different ethnic groups should be carried out with due caution.

Another aspect that should be discussed within the framework of the topic and that has not been explored in this research refers to the technical issues of evaluation of the WC. Although the standardization for obtaining the WC is relevant, the reproducibility of the technique should be an aspect considered in the definition of the protocol for obtaining the measure. External landmarks (minimal waist and umbilicus) are widely used and may be more reproducible as they require less experience from the evaluator. Notwithstanding, the adoption of internal bony landmarks (iliac crest and lowest rib) as a reference shows advantages in the clinical follow-up of measurements, since they remain unaltered with changing adiposity (16). Thus, these aspects should be considered in future research and in the selection of the best assessment protocol.

It should be noted that the comparison of the performance of six measurement sites and the use of a method considered “gold standard” for quantifying visceral fat are important aspects of the study.

In summary, the magnitude of the WC is influenced by the anatomical site of measurement, particularly in women, indicating the need for standardization of protocols for obtaining the measurement and thus allowing valid comparisons between the studies.

In conclusion, among men, the minimal waist showed better correlation with VAT and with cardiometabolic parameters. In women, the WC seems to be a more accurate indicator of subcutaneous fat than of visceral fat. The findings of this study do not provide clear evidence of the superiority of a single measurement to predict the cardiometabolic risk. It would be important to conduct longitudinal studies involving a larger number of participants to compare the predictive ability of different WC measures for the development of cardiovascular and metabolic disorders.

Therefore, data on the preference of a measurement protocol are still limited and more studies need to be developed in order to outline more conclusive evidence on the anatomical site of measurement that should be adopted as a clinical tool to assess the cardiometabolic risk. Replicating this approach in different populations will facilitate global comparisons.
